# Association of Hemorrhoid Vascular Injuries with Cigarette Smoking—An Evaluation with Interesting Prospects

**DOI:** 10.1055/s-0039-1700497

**Published:** 2019-11-07

**Authors:** Savitha V. Nagaraj, Amit Mori, Madhavi Reddy

**Affiliations:** 1 Department of Internal Medicine, The Brooklyn Hospital Center, Brooklyn, New York; 2 Department of Gastroenterology, Center for Digestive Disease, Shenandoah, Texas; 3 Department of Gastroenterology, The Brooklyn Hospital Center, Brooklyn, New York

**Keywords:** hemorrhoids, piles, hemorrhoid vascular injuries, gastrointestinal vasculature, symptomatic hemorrhoids, anal canal, varicose veins of the anus, cigarette smoking, smoking associated vascular injuries

## Abstract

**Background** Hemorrhoids are vascular structures in the anal canal which are seldom used to evaluate vascular diseases. Cigarette smoking is well-known to cause both arterial and venous vascular injuries. However, the impact of smoking on hemorrhoid vasculature is unknown.

**Objective** Considering that vasculature in the hemorrhoids has the same anatomy and pathophysiology of vascular damage as other systemic vasculatures, we conducted this study to evaluate the relation between smoking and incidence of hemorrhoidal vascular injury.

**Design and Data Analysis** Retrospective review of all the screening colonoscopies performed at our Department of Gastroenterology (predominantly serving urban minority population) over 3 years was conducted and patients with recorded smoking history were included in the study ( *n* = 242). Fisher's exact test with two-tailed *p* -value and odds ratio were used to evaluate for the association between smoking and incidence of hemorrhoids.

**Results** We studied 242 subjects and found statistically significant association between smoking and hemorrhoids ( *p* < 0.05) with the risk of developing hemorrhoids among smokers being 2.4 times that of a nonsmoker. We further noted no significant difference in the incidence of hemorrhoidal vascular injuries between the past versus current smokers and male versus female smokers.

**Conclusion** This is one of the first studies to establish an association between smoking and hemorrhoids. Our study shows that the hemorrhoidal vasculature is impacted by smoking similar to other vascular systems. This study sheds light on the possibility of evaluating hemorrhoids for clues of other systemic and gastrointestinal vascular damage. This correlation can add clinical value especially given the flexibility of assessing hemorrhoids as an outpatient in a cost effective and comfortable manner.

Hemorrhoids are an integral part of the gastrointestinal tract and are comprised of both vascular structures and nonvascular supporting structures. Anatomically located in the anal canal, these structures are accessible and can be comfortably examined as an outpatient. Despite these facts, hemorrhoids have not been used to evaluate vascular diseases and are seldom examined in the clinical setting unless symptomatic. It is currently unknown whether hemorrhoidal vascular injury holds a clue to ongoing gastrointestinal vascular injury or other systemic vascular injuries.

Symptomatic hemorrhoids or hemorrhoidal disease, commonly referred to as “hemorrhoids” or “piles” by the general population, are symptomatic enlargement and distal displacement of the anal cushions. One of the commonest classification used to grade hemorrhoids is the Goligher classification where hemorrhoids are graded based on their appearance and degree of prolapse. 1 Grade 1 or first-degree hemorrhoids: anal cushions bleed but do not prolapse; Grade 2 or second-degree hemorrhoids: anal cushions prolapse through the anus on straining but reduce spontaneously; Grade 3 or third-degree hemorrhoids: anal cushions prolapse through the anus on strain and need manual reduction into the anal canal; Grade 4 or fourth-degree hemorrhoids: prolapse is irreducible. Grade 4 also includes thrombosed, incarcerated, and hemorrhoids with circumferential rectal mucosal prolapse. The commonest method to view hemorrhoids has been anoscope, however, hemorrhoids can also be well assessed by intrarectal retroflexion of the colonoscope or transparent anoscope with flexible endoscope. 2 To assess hemorrhoids during colonoscopy, degree of the mucosal elevation of rectal columns, changes in color including the existence and degree of red color sign, dilated vein and, white area, and the existence and size of hypertrophied anal papillae are evaluated. 3

Many studies have tried to understand the mechanism of development of hemorrhoids and have proposed different theories regarding the development of hemorrhoidal disease. One of these theories is of sliding anal canal in which anal canal disintegration is believed to result in venous dilation. 4 Other studies attribute hemorrhoidal disease to vascular injury of the hemorrhoid plexus with or without anal canal disintegration. Irrespective of whether the anal cushion disintegration caused the venous dilation or venous dilation occurred initially, the resultant common vascular injuries include venous swelling, engorgement, and clot formation. One of the studies evaluated over a hundred histological specimens of hemorrhoidectomy and noticed severe inflammatory reaction affecting mainly the blood vessel wall and the surrounding connective tissue. 5 This finding showed that the dysregulation of vasculature could have contributed to symptoms of hemorrhoidal disease by causing ischemia, thrombosis, and ultimately venous dilation and engorgement of the hemorrhoidal veins. 5 6

Vascular smooth muscle is well known to be regulated by cytokines, endothelium, enzymes, and hormones. An imbalance in the endothelium-derived relaxing factors such as prostacyclin, nitric oxide, and endothelium-derived constricting factors such as endothelin and reactive oxygen free radicals has been implicated in vascular disorders. 7 One of the studies has shown evidence of statistically significant increase in microvascular density, vascular endothelial growth factor, matrix metalloproteinase 9, and nitric oxide synthase on the internal hemorrhoid tissue compared with normal anal cushion. 8 This suggested involvement of inflammatory factors in the pathogenesis of hemorrhoids and angiogenesis as a one of the possible mechanisms of vascular injury in hemorrhoids. 8 Cigarette smoking has been known to cause such effects on vasculature. 6

Smoking has been found to cause vascular injuries by direct endothelial damage through the release of carbon monoxide, along with other mechanisms of increasing free radicals, oxidative stress, and release of inflammatory cytokines. 6 Cigarette smoking is a well-known risk factor for vascular damage and is implicated in numerous vascular diseases such as atherosclerosis, peripheral vascular disease, coronary artery disease, abdominal aortic aneurysm, and varicose veins. When the relationship between smoking and major arterial and venous systems has been well established, it is intriguing to evaluate for similar effects of vascular injury, if any, on the hemorrhoidal vasculature.

In this study, we hypothesized that hemorrhoidal vasculature may be affected by smoking and tried to study its association with cigarette smoking. An implication if found, can open up new prospects to study correlation between hemorrhoidal vascular injuries and other gastrointestinal and systemic vascular injuries. This can further be used to develop ways to use hemorrhoidal vasculature to evaluate other vascular systems in a cost effective and minimally invasive method in an outpatient setting.

## Study Method

We conducted a retrospective case-control study at our institution where all the colonoscopies performed in the department of gastroenterology over a span of 3 years between 2012 and 2015 were reviewed. The original observational study protocol was approved by our institutional review board and this study was conducted using the information acquired from the original data pool as a secondary study. For the purpose of this study, all the patients who had colonoscopies for screening purposes as an outpatient and recorded smoking history were included. Patients who had colonoscopies performed for reasons other than screening or as an inpatient and/or whose smoking history was unavailable, were excluded from the study.

Patient's chart and colonoscopy reports were reviewed. Data including demographic information such as age, sex, ethnicity, body mass index (BMI), social history including smoking history with special emphasis on the duration of smoking, past versus current smoking were obtained. Hemorrhoids were identified during colonoscopy by intrarectal retroflexion and forward view by evaluating for degree of mucosal elevation of rectal columns, changes in color including the existence and degree of red color sign, dilated vein and white area, and the existence and size of hypertrophied anal papillae. Goligher classification of hemorrhoids was not used for reporting of all the identified hemorrhoids. Colonoscopy reports were reviewed for the diagnosis of hemorrhoids.

Case control study was conducted, and patients were divided into two groups: Group A—current and former smokers; Group B—lifetime nonsmokers. Descriptive data analysis was performed. Incidence of hemorrhoids in both the groups was calculated and the data were analyzed for the association between smoking and hemorrhoids using Fisher's exact test with two-tailed *p* -value. Odds ratio was calculated with 95% confidence interval for relative risk of hemorrhoids in the smokers group compared with nonsmokers group. The statistical significance level was set at *p* < 0.05.

## Results

**Fig. 1:**
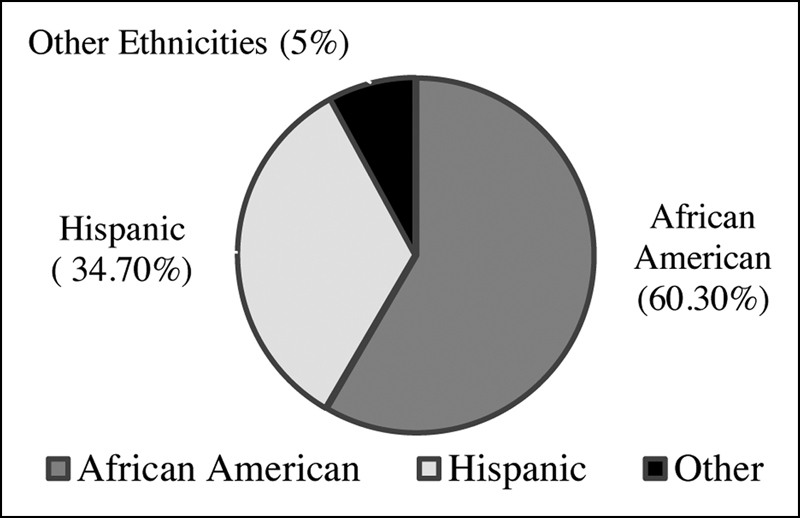
Pie-graph showing distribution of ethnicities. Fig. 1 depicts the distribution of ethnicities. Majority of our study population were African American 60.3% (146/242), followed by Hispanics 34.7% (84/242) and other ethnic groups 5% (12/242).

The study included a total of 242 patients who underwent screening colonoscopies as an outpatient and had recorded data of their smoking history. Majority of the study population were African American 60.3% (146/242), followed by Hispanics 34.7% (84/242) and other ethnic groups 5% (12/242) ( Fig. 1 ). The total number of males (20% [49/242]) were lower compared with females (80% [193/242]) in our study population. Among the males, majority were smokers (53% [26/49]) and among the females, majority were nonsmokers (68% [132/193]).

The patients were divided into two groups: Group A—smokers and Group B—nonsmokers. Group A comprised of approximately 36% (87/242) of the study population who were former smokers (44.8%) or current smokers (55.2%); Group B comprised of approximately 64% (155/242) of the study population who were lifetime nonsmokers. Average age of subjects in Group A was 57 years and Group B was 59 years. Average BMI of Group A was 30.7 and Group B was 30.9. This comparison between smokers and nonsmokers is displayed in Table 1 .

**Table 1:** Comparison of demographic data between Group A (smokers) and Group B (nonsmokers)

Parameters of comparison	Group A: smokers ( *N* = 87)	Group B: nonsmokers ( *N* = 155)
**Average age (y)**	57	59
**Average BMI**	30.7	30.9
**Male (** ***n*** ** = 49)**	26	23
**Female (** ***n*** ** = 193)**	61	132

Note: Table 1 displays the comparison of age, sex, and body mass index (BMI) between the two study groups: smokers: Group A and nonsmokers: Group B. The average age and BMI are similar in both the groups. The number of male smokers is higher than male nonsmokers, whereas, the number of female nonsmokers is higher than female smokers.

**Fig. 2:**
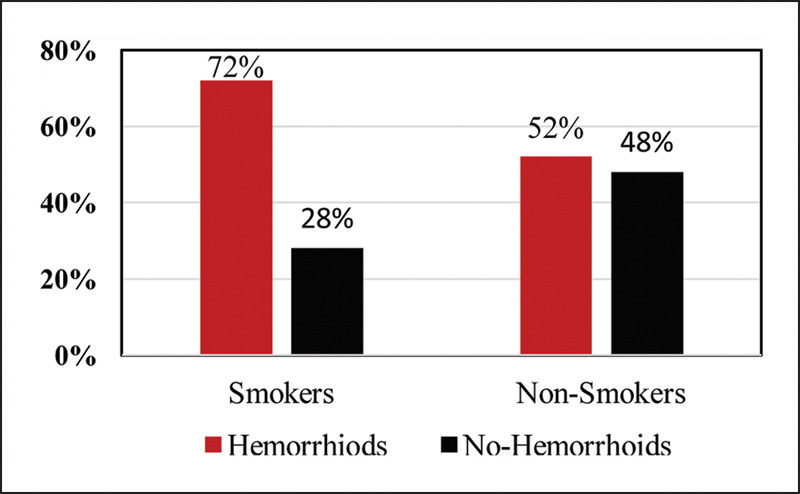
Comparison of incidence of hemorrhoids between Group A (smokers) and Group B (nonsmokers). Fig. 2 is a graphical representation of the incidence of hemorrhoids which were significantly higher among the smokers (Group A) with 72% of the population having colonoscopy findings of hemorrhoids when compared with the nonsmokers (Group B) with 52% having findings of hemorrhoids.

The incidence of hemorrhoids was significantly higher among the smokers (Group A) with 72% of the population having colonoscopic findings of hemorrhoids when compared with the nonsmokers (Group B) with 52% having findings of hemorrhoid vascular changes ( Fig. 2 ). Fisher's exact test with two-tailed *p* -value calculation showed strong association between smoking and hemorrhoids with *p* = 0.0026 indicating the association as very statistically significant. The odds ratio was 2.4 with 95% confidence interval of 1.36 to 4.22 and *p* = 0.0025.

Further analysis of Group A or smokers group showed a decrease in the incidence of hemorrhoids among past smokers (79%) compared with the current smokers (81%) but the difference was not statistically significant ( *p* = 1.0). Male and female smokers when compared did not have significant difference in the incidence of hemorrhoids (81 and 80%, respectively). However, incidence of hemorrhoids was higher among female nonsmokers (54%) compared with the male nonsmokers (43%). These findings are shown in Table 2 .

**Table 2:** Comparison of incidence of hemorrhoids between Group A (smokers) and Group B (nonsmokers) and subgroups of Group A and Group B

Study groups	Subgroups	Sample size ( *N* )	Hemorrhoids ( *N* [%])	Nonhemorrhoids ( *N* [%])
**Smokers (Group A)**	Total	87	63 (72%)	24 (28%)
	Past	39	31 (79%)	8 (21%)
	Current	48	39 (81%)	9 (19%)
	Male	26	21 (81%)	5 (19%)
	Female	61	49 (80%)	12 (20%)
**Nonsmokers (Group B)**	Total	155	81 (52%)	74 (48%)
	Male	23	10 (43%)	13 (57%)
	Female	132	71 (54%)	61 (46%)

Note: Table 2 compares the incidence of hemorrhoids among the study groups. We notice that the incidence of hemorrhoids was significantly higher among the smokers (Group A)—72% compared with nonsmokers (Group B)—52%. Further analysis of Group A or smokers group showed a decrease in the incidence of hemorrhoids among past smokers (79%) compared with the current smokers (81%) but the difference was not statistically significant ( *p* = 1.0). Male and female smokers did not have significant difference in the incidence of hemorrhoids (81 and 80%, respectively). However, incidence of hemorrhoids was higher among female nonsmokers (54%) compared with the male nonsmokers (43%).

## Discussion

Hemorrhoids are normal anatomical structures in the anal canal comprising of arterioles, venules, and smooth muscle fibers. Swelling and engorgement of this vasculature in the hemorrhoid plexus result in acute painful swelling of the hemorrhoid, frequently referred to as “varicose veins of the anus,” “piles,” or simply “hemorrhoids.” Progressive injury to hemorrhoidal vasculature leads to the symptoms of pain, prolapse, and bleeding.

There are different theories regarding the pathology of development of symptomatic hemorrhoids, one of which is the theory of sliding anal canal which proposes that the hemorrhoids occur due to the disintegration or deterioration of anal cushions which in return causes slippage and hence venous dilation. 4 However, there are other theories which show that symptomatic hemorrhoids occur as a result of injury to the vascular structures in the hemorrhoids with or without injury to the other structures in the anal cushions. For the relevance of this study, discussion will be focused on injuries to the hemorrhoidal vasculature.

Our study showed a very statistically significant association between the history of smoking and incidence of hemorrhoids. The risk of developing hemorrhoidal disease among smokers was 2.4 times that of a nonsmoker ( *p* < 0.05). We did not note a statistically significant difference in the incidence of hemorrhoids between past and current smokers, which leads us to evaluate whether these vascular injuries already caused by smoking are difficult to reverse once smoking is discontinued. The BMI and age of both smokers and nonsmokers in our study population were similar. This shows that the increased risk of hemorrhoids was predominantly secondary to smoking associated vascular damage rather than other causes such as obesity or related constipation in our study population.

The highest ethnic population was of African American followed by Hispanics. However, this ethnicity distribution can also be attributed to the location of our institution and the population served by our hospital which is predominantly urban minority populations such as African American, Hispanic and Asian. In our study, we noted a higher number of smokers compared with nonsmokers in the male population, whereas majority of the females were nonsmokers. The incidence of hemorrhoids, however, was similar among both male and female smokers (higher than nonsmokers in both populations). In the nonsmokers group, the incidence of hemorrhoids was higher among females compared with males, which is likely due to factors causing increased intra-abdominal pressure such as pregnancy and related straining and constipation.

The limitations of our study include the lack of documentation of grading of hemorrhoids and the diagnosis of hemorrhoids being based on the gross appearance during colonoscopy. In addition, no documentation of histopathological analysis of the hemorrhoids to evaluate for vascular inflammation was available. The above mentioned limitations are predominantly due to the retrospective nature of our study that relied mainly on clinical documentation.

Cigarette smoking has long been associated with both arterial and venous vascular injuries. Smoking has been well implicated in increasing the risk of both venous and arterial vascular diseases such as coronary artery disease, stroke, Buerger's disease, varicose veins, cerebral and abdominal aneurysms to name a few. Framingham study showed an increase in the incidence of varicose veins among smokers when compared with the nonsmokers. 9 Smoking has been known to cause these vascular injuries by many mechanisms, some of them being oxidative stress and hypoxia. Hypoxia leads to precapillary closure of sphincters resulting in venous hyperpressure ultimately leading to weakening of the sphincter causing varicose veins. 10 11 Similar such sphincter findings have been seen in hemorrhoidal vasculature. Hypoxia is also known to activate endothelial cells, leading to increased proinflammatory factors in the vessel wall causing inflammation and injury, 10 11 a pathology similar to the hemorrhoids. 12 Recent studies have shown association between varicose veins and coronary artery ectasias as well as hemorrhoids and coronary artery disease. 12 13 14 15

Looking at these similarities in the pathology and clinical presentation of vascular injuries in the varicose veins and hemorrhoidal veins, it is likely that hemorrhoids face the same effect of vascular damage from smoking as the systemic vasculature and the lower extremity varicose veins. It is also possible that hemorrhoidal vascular damage is accompanied by other vascular (arterial and venous) injuries. This association if further proven, hemorrhoid vasculature may be evaluated to monitor for systemic vascular injuries including gastrointestinal vascular injuries.

## Conclusion

This is one of the first studies showing the effect of smoking on hemorrhoidal vasculature. In this study, we found statistically significant association between smoking and hemorrhoids with the risk of developing hemorrhoid vascular injuries among smokers being 2.4 times that of nonsmokers. We further noted no significant difference between the incidence of hemorrhoidal vascular injuries between male and female smokers and past and current smokers.

Our study shows that hemorrhoidal vasculature, a part of gastrointestinal vasculature, is prone to vascular damage and impacted by smoking like any other vascular system. This information can be further used to evaluate whether hemorrhoids can be used to assess and monitor for arterial and venous vascular injuries and smoking-related vascular injuries.

Compared with other vascular systems, one of the benefits of hemorrhoidal vasculature is owing to its anatomical location which can be accessed effectively and comfortably in an outpatient setting. The other benefit is considering the size of the hemorrhoidal vasculature, vascular damages may be visible in the earlier phases of injury. Further research is however, needed to develop the best method to effectively examine and assess hemorrhoids as an outpatient and to evaluate its association with various other vascular injuries.

## Notes

**Erratum:** Fig. 1 of this article has been updated as per Erratum published on January 14, 2020.
