# Erratum: Association of Hemorrhoid Vascular Injuries with Cigarette Smoking—An Evaluation with Interesting Prospects

**DOI:** 10.1055/s-0040-1701247

**Published:** 2020-01-14

**Authors:** Savitha V. Nagaraj, Amit Mori, Madhavi Reddy

**Affiliations:** 1 Department of Internal Medicine, The Brooklyn Hospital Center, Brooklyn, New York; 2 Department of Gastroenterology, Center for Digestive Disease, Shenandoah, Texas; 3 Department of Gastroenterology, The Brooklyn Hospital Center, Brooklyn, New York

It has been brought to our attention that Figure 1 was incorrect in the above article published online in Volume 5, Issue 4 of *The Surgery Journal* ( 10.1055/s-0039-1700497 ). The correct figure has now been updated as given below.

**Fig. 1:**
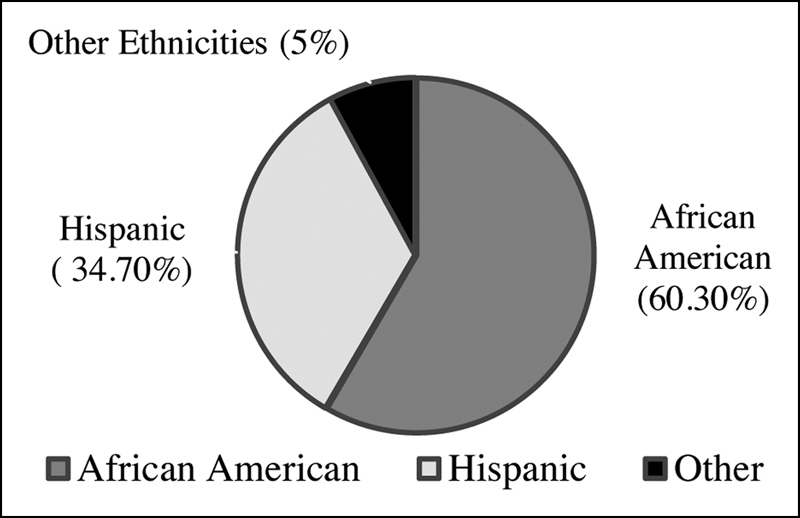
Pie-graph showing distribution of ethnicities. Fig. 1 depicts the distribution of ethnicities. Majority of our study population were African American 60.3% (146/242), followed by Hispanics 34.7% (84/242) and other ethnic groups 5% (12/242).

